# A Speech-Level–Based Segmented Model to Decode the Dynamic Auditory Attention States in the Competing Speaker Scenes

**DOI:** 10.3389/fnins.2021.760611

**Published:** 2022-02-10

**Authors:** Lei Wang, Yihan Wang, Zhixing Liu, Ed X. Wu, Fei Chen

**Affiliations:** ^1^Department of Electrical and Electronic Engineering, Southern University of Science and Technology, Shenzhen, China; ^2^Department of Electrical and Electronic Engineering, The University of Hong Kong, Pokfulam, Hong Kong SAR, China

**Keywords:** auditory attention decoding, speech-RMS-level segments, auditory attention switching, temporal response function, EEG signals

## Abstract

In the competing speaker environments, human listeners need to focus or switch their auditory attention according to dynamic intentions. The reliable cortical tracking ability to the speech envelope is an effective feature for decoding the target speech from the neural signals. Moreover, previous studies revealed that the root mean square (RMS)–level–based speech segmentation made a great contribution to the target speech perception with the modulation of sustained auditory attention. This study further investigated the effect of the RMS-level–based speech segmentation on the auditory attention decoding (AAD) performance with both sustained and switched attention in the competing speaker auditory scenes. Objective biomarkers derived from the cortical activities were also developed to index the dynamic auditory attention states. In the current study, subjects were asked to concentrate or switch their attention between two competing speaker streams. The neural responses to the higher- and lower-RMS-level speech segments were analyzed *via* the linear temporal response function (TRF) before and after the attention switching from one to the other speaker stream. Furthermore, the AAD performance decoded by the unified TRF decoding model was compared to that by the speech-RMS-level–based segmented decoding model with the dynamic change of the auditory attention states. The results showed that the weight of the typical TRF component approximately 100-ms time lag was sensitive to the switching of the auditory attention. Compared to the unified AAD model, the segmented AAD model improved attention decoding performance under both the sustained and switched auditory attention modulations in a wide range of signal-to-masker ratios (SMRs). In the competing speaker scenes, the TRF weight and AAD accuracy could be used as effective indicators to detect the changes of the auditory attention. In addition, with a wide range of SMRs (i.e., from 6 to –6 dB in this study), the segmented AAD model showed the robust decoding performance even with short decision window length, suggesting that this speech-RMS-level–based model has the potential to decode dynamic attention states in the realistic auditory scenarios.

## Introduction

In a competing speaker environment, the target speech perception relies on the modulation of selective auditory attention. A large number of behavioral and neuroimaging studies have investigated the human abilities to selectively track the particular speech stream with sustained auditory attention (e.g., Cherry, 1953; Shamma and Micheyl, 2010; Szabó et al., 2016). Nevertheless, the dynamic change of the auditory attention states often occurs in the real-life environments, which requires the auditory system to reorganize the relevant information of specific auditory objects and reallocate attention resources when the focus of attention switches between different speaker streams (e.g., Fritz et al., 2007, 2013; Ahveninen et al., 2013). Some studies also suggested that, in the dynamic auditory scenes, the salient speech features played an important role in the target speech perception through the bottom-up auditory pathways (Kaya and Elhilali, 2014; Shuai and Elhilali, 2014). However, it remains unknown whether the dynamic change of the auditory attention states can be reliably decoded from the cortical signals when subjects focus their attention to the natural sentences in the complex auditory scenes. Besides, it needs to further uncover the underlying neural mechanisms of the sensitive tracking ability to the target speech stream in the complex auditory scenes.

Several methods have been proposed to detect selective auditory attention on the basis of the typical electroencephalograph (EEG) features with diverse experimental tasks (e.g., Näätänen et al., 1992; Choi et al., 2013; Larson and Lee, 2014; Geravanchizadeh and Roushan, 2021). In earlier electrophysiological studies, the dynamic states of the auditory attention were captured by comparing the morphology of event-related potential (ERP) components (e.g., the P1–N1–P2 complex, P300) elicited by the acoustic properties within different auditory stimuli (e.g., Polich et al., 1986; Tse et al., 2004; Choi et al., 2013). Although such ERP-based measurements were extensively used in the brain–computer interface speller system (e.g., Donchin et al., 2000; Hoffmann et al., 2008), it was an inappropriate method for detecting the dynamic attention changes in the continuous natural speech streams. Recently, some researchers further developed proper experimental paradigms and analytical methods to explore the dynamic switching of the auditory attention under the multi-talker conditions using the EEG signals (e.g., Lee et al., 2014; Deng et al., 2019; Geirnaert et al., 2020; Getzmann et al., 2020). Specifically, two typical characteristics of EEG signals, i.e., the stronger N2 subcomponent and the lateralization of posterior alpha power, were significantly correlated with the spatial auditory attention switching (e.g., Deng et al., 2019; Getzmann et al., 2020). Nevertheless, these ERP-based features required average cortical responses over multiple experimental trials to obtain the high-quality time-locked characteristics. Hence, because of the time-consuming process of extracting attention-related features, these ERP-based methods were limited to be used in the realistic auditory scenes. Many studies also used common spatial patterns and effective connectivity to decode the dynamic attention states in single-trial EEG signals when subjects performed the dichotic listening tasks (e.g., Geirnaert et al., 2020; Geravanchizadeh and Gavgani, 2020). The spatial differences among speakers evoked distinct brain activity patterns and such features provided crucial cues to decode the selective auditory attention. However, in the absence of spatial cues, there was little understanding about the effect of dynamic attention modulation on the target speech perception in the multi-speaker conditions.

The recent understanding of the selective auditory attention in the cocktail party problem and the advances of electrophysiological technologies make it possible to decode the auditory attention from EEG signals in the complex auditory scenarios. In the natural continuous speech streams, the extensively used auditory attention decoding (AAD) methods were based on the mapping functions between the speech envelope and the corresponding EEG responses *via* linear and non-linear computational models (e.g., Ding and Simon, 2012b; O’Sullivan et al., 2015; Crosse et al., 2016; Ciccarelli et al., 2019; Das et al., 2020; Geravanchizadeh and Roushan, 2021). Specifically, the linear decoder models, such as the temporal response function (TRF), were widely used to decode auditory attention with reasonable accuracy under a wide range of signal-to-masker ratios (SMRs) (Crosse et al., 2016). Generally, the estimation procedure of linear models was simpler and faster than that of non-linear models. The linear models also provided the interpretable relations between the continuous auditory stimulus and the corresponding EEG responses (e.g., Ding and Simon, 2012b; O’Sullivan et al., 2015). The non-linear decoding models using deep neural networks (DNNs) can achieve higher AAD accuracies compared to the linear AAD approaches even with short decoding window lengths (e.g., Ciccarelli et al., 2019; Das et al., 2020). Nevertheless, it was still difficult to interpret the underlying mechanisms for the decoding results by the DNN-based models. Besides, most non-linear decoding models concentrated on feature extraction from EEG signals but ignored the features carried by speech temporal envelopes. Briefly, these effective AAD methods have successfully decoded the auditory attention when subjects kept their attention to a specific target stream throughout the experimental procedure. Several magnetoencephalography and EEG studies also indicated that the AAD methods could track the dynamic changes of attentional states when the competing speakers were presented at the same or different spatial locations (e.g., Akram et al., 2016; Miran et al., 2018, 2020; Teoh and Lalor, 2019). Nevertheless, it remains unclear how the neural responses are affected by the dynamic change of attention states and which speech features make great contributions to capturing changes in auditory attention states (i.e., before or after the auditory attention switching) in the absence of the spatial cues between the competing speakers under different SMR conditions.

In general, selective auditory attention can realize successful perception of the target auditory object by activating the target-related information and inhibiting the irrelevant information (Fritz et al., 2007; Shamma and Micheyl, 2010; Szabó et al., 2016). The target speech perception in noise depends on the robust representation regions of the target signal and the regions that are least affected by the competing speaker stream (Cooke, 2006; Li and Loizou, 2007). Specifically, in the competing speaker environments, the salient auditory cues and silent gaps of the auditory stimuli play an important role in target speech perception (e.g., Li and Loizou, 2007; Vestergaard et al., 2011; Seibold et al., 2018). The speech temporal information at low frequency containing the syllable rhythms can also facilitate target speech perception in noisy conditions (e.g., Greenberg et al., 2003; Vestergaard et al., 2011). As indicated in the investigations from previous studies (e.g., Kates and Arehart, 2005; Chen and Loizou, 2012; Chen and Wong, 2013), speech envelopes not only revealed the change of relative root mean square (RMS) intensity but also conveyed the phonetic distribution of the whole sentences. The analysis of different speech segments on the basis of relative RMS intensity provided an effective way to understand the attentional modulation of target speech perception in the competing speaker environments (Chen and Loizou, 2011; Wang et al., 2020a,b). According to previous studies, the higher- and lower-RMS-level speech segments could be extracted with a threshold of –10 dB relative to the overall RMS level of the speech signal (e.g., Kates and Arehart, 2005; Chen and Wong, 2013). Higher-RMS-level speech segments contained the voicing parts of the sentences (i.e., the most proportion of vowels and vowel–consonant transitions), whereas most silent gaps and weak consonants were located in lower-RMS-level speech segments (Chen and Loizou, 2011; Chen and Wong, 2013). Previous studies also demonstrated that higher- and lower-RMS-level–based speech segments had different effects on the encoding and decoding of the target speech from the corresponding EEG signals (Wang et al., 2019, 2020a,b). Moreover, in cases where the listeners were required to maintain their attention on the target speech stream, the AAD sensitivity and accuracy could be improved by using the time-variant segmented model to decode different types of RMS-level–based speech segments (Wang, 2021). Accordingly, it is valuable to further explore whether the speech-RMS-level–based segmented AAD model could reliably track the dynamic change of the auditory attention states in the competing speaker scenes. The contribution of different RMS-level–based speech segments on attention decoding needs to be studied in the auditory attentional switching tasks, so as to expand the potential application of the neurofeedback-based AAD system in the realistic auditory scenarios.

In the present study, we hypothesized that effective biomarkers can be extracted from the cortical responses to index the dynamic auditory attention states in the competing speaker scenes with a wide range of SMRs. Furthermore, RMS-level–dependent speech segmentation would have a significant influence on the decoding performance of selective auditory attention. Hence, the speech-RMS-level–based segmented model could have the potential to improve the AAD accuracy and sensitivity with both the sustained and switched auditory attention modulations. In addition, the auditory attention states and the relative SMR levels could jointly affect the AAD abilities in the competing speaker scenes.

## Materials and Methods

### Participants

Sixteen participants (10 males and 6 females) aged between 16 and 27 years old participated in this experiment. All participants had normal hearing abilities with the pure-tone threshold less than 25 dB at 125–8,000 Hz. All subjects were native speakers of Mandarin Chinese and provided informed written consent before their participations. The Institution’s Ethical Review Board of Southern University of Science and Technology approved the experimental procedures.

### Stimuli and Experimental Procedure

**FIGURE 1:**
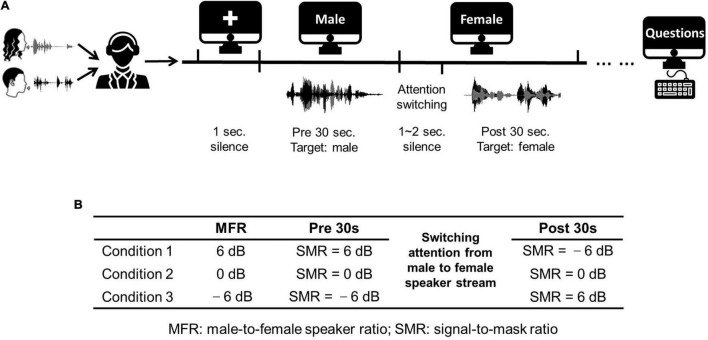
**(A)** An illustration of the experimental procedure. **(B)** Three conditions used in this study. Three conditions fixed the MFR levels at 6, 0, and –6 dB, and the SMR level were changed in the first half and the latter half with the switching of the auditory attention from the male to the female speaker streams. The icons used in **(A)** obtained from https://thenounproject.com/.

The stimuli used in this work were extracted from two Chinese stories narrated by a female Mandarin speaker and a male Mandarin speaker. These stories were divided into approximately 60-s segments. Each experimental trial contained a 60-s speech fragment. The silent gaps within each 60-s fragment were less than 300 ms to avoid unexpected auditory attention shifts. To test the neural responses with the switching of attention, subjects were required to shift their attention from the male speaker to the female speaker at the middle time of each 60-s segment. Hence, the auditory attention switching divided the whole trial into two different sections (i.e., the first half and the latter half). Specifically, each trial contained a 30-s speech fragment with the attention to the male speaker in the first half, followed with a silent gap with random duration (1∼2 s), and a 30-s speech fragment with the attention to the female speaker in the latter half. Figure 1A displays the detailed experimental procedure. The male-to-female ratio (MFR) was fixed in each condition, and there were three MFR conditions (i.e., 6, 0, and –6 dB) in this study. More specifically, for the conditions at 6- and –6-dB MFR levels, the SMR level was changed with the switching of attention from the male to the female speaker stream, whereas the SMR level was unchanged before and after the switching of the auditory attention for the 0-dB MFR condition. The detailed experimental settings about the three MFR conditions are shown in Figure 1B. During the whole experiment, visual instructions were displayed on the screen to control the experimental procedure. The visual instructions were represented on the screen with white color against the black background. In each trial, a white cross was displayed in the middle of the screen without auditory stimuli. Then, the character “male” appeared on the screen to remind the listener to focus on the male speaker stream. Subsequently, the instruction on the screen was changed to “female” to remind the listener to switch his/her attention to the female speech stream. To avoid the influence of visual changes on the neural responses, the auditory stimuli in the second stage played 1∼2 s after the change of visual instruction. Each trial was played once to each subject. Five trials were included in each block. At the end of each block, three questions about the target speech streams with four choices were asked to the participant. The block with all corrected answers was reserved for further analyses. Two blocks (i.e., 10 trials) were obtained for each condition.

The experiment was performed in a double-walled acoustically shielded room. Mixed auditory stimuli were presented bilaterally *via* earphones at 65-dB sound pressure level. The whole experimental procedures were controlled by the software E-Prime 2. This experiment used 62 electrodes to record the scalp EEG signals at the 500-Hz sampling rate. Two external reference electrodes were placed at the left and right mastoids. An online reference electrode was attached at the nose tip, and the electrooculography signals were recorded by two electrodes located below and up the left eye. The impedance of all EEG electrodes was kept less than 5 kΩ. During the experiment, all participants were required to reduce body movements.

### Data Analyses

#### Electroencephalograph Signals and Auditory Stimuli Preprocessing

The preprocessing of the EEG signals was conducted with the EEGLAB toolbox (Delorme and Makeig, 2004). First, a high-pass filter with the cutoff frequency of 0.5 Hz was implemented with the function of windowed sinc finite impulse response (FIR) filter in the EEGLAB toolbox. Independent component analysis was implemented to remove typical artifacts (e.g., eye movements) using the ICLabel toolbox (Pion-Tonachini et al., 2019). On average, three independent components were removed for each subject. The EEG signals were then filtered at low-frequency bands because the cortical responses at these low frequencies could reliably track the speech envelopes (e.g., Di Liberto et al., 2015; O’Sullivan et al., 2015; Wang et al., 2019). Specifically, the EEG signals were high-pass filtered with a zero-phase FIR filter at a cutoff frequency of 2 Hz and low-pass filtered with a zero-phase FIR filter at a cutoff frequency of 8 Hz.

**FIGURE 2:**
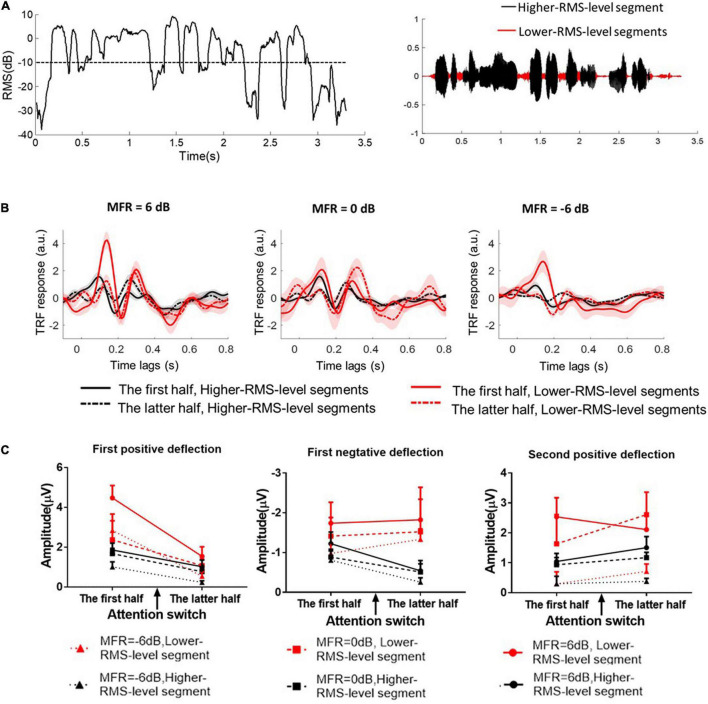
**(A)** The root mean square (RMS) level of a short fragment in the continuous speech stimulus. The dashed line indicates the threshold (–10 dB relative RMS level) to classify higher- and lower-RMS-level speech segments within the continuous utterance. The right figure shows the higher- and lower-RMS-level segments in the temporal series of a sentence. **(B)** The TRF responses calculated between speech envelopes and the corresponding EEG signals in 6–, 0–, and –6-dB MFR conditions. The TRF responses between higher-RMS-level speech segments and EEG signals were displayed with the black solid line in the first half (i.e., before attention switching) and with the black dashed line in the latter half (i.e., after attention switching) of the whole trial. The right solid line in the first half (i.e., before attention switching) and the right dashed line in the latter half (i.e., after attention switching) of the whole trial were represented the TRF responses derived from lower-RMS-level speech segments and the corresponding EEG signals, respectively. **(C)** The amplitudes of the three typical TRF components with the lower-RMS-level segments (right lines) and higher-RMS-level segments (black lines) in –6 dB (dot lines with the rectangular sign), 0 dB (dashed lines with the square sign), and 6 dB (solid lines with the circle sign) before and after the switching of the auditory attention from the male speaker (the first half) to the female speaker (the latter half).

Speech envelopes were extracted as the primary feature to calculate the cortical tracking ability (e.g., O’Sullivan et al., 2015; Crosse et al., 2016; Das et al., 2020). This study further investigated the effects of RMS-level–based segmentation on the phase-locking performance between cortical responses and speech envelopes at low frequencies. First, speech signals were divided into the higher- and lower-RMS-level–based segments on the basis of the threshold of –10 dB relative to the overall RMS level of the whole utterance. The detailed segmentation procedures can also refer to Kates and Arehart (2005) and Wang (2021). Figure 2A shows the RMS level of a continuous utterance and higher- and lower-RMS-level segments within this sentence. This segmentation threshold (i.e., –10 dB relative to the RMS level of the whole sentence) was determined according to the distribution of perceptual information in different RMS-level–based speech segments, which was originally proposed in Kates and Arehart (2005) and extensively studied in many behavioral and electrophysiological experiments (e.g., Kates and Arehart, 2005; Chen and Loizou, 2011, 2012; Chen and Wong, 2013; Wang et al., 2019, 2020a,b; Wang, 2021). Previous studies have found that higher-RMS-level–speech segments mainly contained the vowels and transitions between vowels and consonants, whereas lower-RMS-level speech segments carried the weak consonants and silent gaps of the continuous utterance (Chen and Loizou, 2011, 2012; Chen and Wong, 2013). In Mandarin sentences, most voicing parts of the whole sentence were in higher-RMS-level speech segments, which contained the vital speech intelligibility information (Chen and Loizou, 2011; Wang et al., 2020b). Some syllabic onsets and the silences of the continuous Mandarin sentences were primarily contained in lower-RMS-level speech segments, which carried the dynamic temporal structure of target speech in noisy conditions (Fogerty and Kewley-Port, 2009; Hamilton et al., 2018). Subsequently, speech envelopes were calculated using the Hilbert transform function in higher- and lower-RMS-level speech segments, respectively. Because the envelope onsets made great contributions to the neural-speech tracking performance (e.g., Hamilton et al., 2018), speech envelopes were then half-wave rectified and the first-order derivative was calculated to extract the increased envelope fluctuations (i.e., the positive derivate values). Then, speech envelopes were resampled to the EEG sampling rate (i.e., 500 Hz) and filtered band-pass filtered from 2 to 8 Hz using the zero-shifted FIR. To reduce the processing time, the processed EEG and speech signals were then downsampled at the sampling rate of 100 Hz.

#### Forward Temporal Response Function Models and Neural Response Predictions

The relationships between speech envelopes and the corresponding EEG activities were analyzed with the linear TRF model using the mTRF toolbox (Crosse et al., 2016). The forward TRF was used to map the cortical responses elicited by the continuous speech stimuli. In this study, how cortical activity encoded different segments in the target speech (i.e., higher- and lower-RMS-level speech segments) and attentional switching (i.e., attention switching from one speaker to the other) was analyzed through TRF responses under various MFR conditions (i.e., 6, 0, and –6 dB). Specifically, the linear transformation of the stimulus envelopes *S(t)* to the corresponding cortical responses *R(t)* can be represented by the linear regression model TRF, as


(1) $$R\,\left(t\right)=T\,R\,F^{*}\,S\,\left(t\right),$$

where * indicates the convolution operator. The TRF can be calculated as


(2) $$T\,R\,F={\left(S^{T}\,S+\lambda \,\text{I}\right)}^{-1}\,S^{T}\,R,$$

and the ridge regression is used to prevent overfitting, where *I* is the identity function and λ represents the ridge parameter. The ridge parameter is determined by the minimum mean-square error between the predicted and original neural signals using the leave-one-out cross-validation. The weights in the TRF model indicate the neural responses relative to the auditory stimulus onsets, and the time lags between –100 and 800 ms were used in this work to show the TRF responses under different experimental conditions. The processing step refers to previous studies (e.g., Di Liberto et al., 2015; Wang et al., 2020b) and the detailed descriptions can also be seen in Crosse et al. (2016). The TRF components show similar response patterns as those in ERPs with specific time lags (e.g., Lalor et al., 2009; Kong et al., 2014; Di Liberto et al., 2015). The TRF weights indicate the correlation coefficients between the speech envelope and the corresponding neural response. The TRF polarity represents the relationship between the cortical current directions and the speech envelope fluctuated trends (Ding and Simon, 2012b). In this study, the TRF weights averaged across all electrodes were statistically analyzed in three typical components, i.e., the first positive component (80∼150 ms), the first negative component (170∼240 ms), and the second positive component (250∼350 ms), with higher- and lower-RMS-level speech segments before and after the attention switching between two speaker streams in 6–, 0–, and –6-dB MFR conditions.

#### Higher- and Lower-Root Mean Square-Level Speech Segments Classification

Higher- and lower-RMS-level segments of the target speech streams can be classified with the corresponding EEG signals, according to the different neural response patterns to these speech segments in clean and noisy environments (e.g., Wang et al., 2019, 2020a). The subject-specific support vector machine (SVM) classifier was used to classify higher- and lower-RMS-level speech segments on the basis of the cross-correlations between speech envelopes and neural responses. In the training procedure, binary speech labels were generated to represent higher- and lower-RMS-level segments of the clean target speech. Then, the feature vector of each channel was composed of the maximum cross-correlation values between the EEG signals and the relevant speech envelopes at each short frame. Specifically, the EEG signals and speech envelopes were divided into 400-ms short frames with a 20% overlapping ratio because the cortical activity mainly responded to the auditory stimulus in the time lag interval (from 0 to 400 ms) as shown in the Figure 2B and the related results in previous studies (e.g., Wang et al., 2020b; Wang, 2021). For each subject, the SVM classifier with a Gaussian radial kernel function was trained to predict higher- and lower-RMS-level segments of the target speech stream on the basis of the corresponding EEG signals using the leave-one-out cross-validation approach. During the testing phase, the analyzed features were derived from the maximum cross-correlation coefficients between the EEG signals and the auditory envelopes from mixed speech sources. The trained SVM model and the calculated feature vectors were used to predict higher- and lower-RMS-level segments within the continuous auditory stimuli. The classification accuracies were calculated by the percentage of correctly identified labels relative to the labels of the target speech source before and after the attentional shifts at different SMR conditions. The SVM classification was computed with the functions in the Statistics and Machine Learning Toolbox Release 2017b of MATLAB (MathWorks Inc., United States).

#### Backward Temporal Response Function Methods and Speech Reconstruction

The backward linear TRF models were widely used in decoding of the auditory attention under the competing speaker environments. The envelope of the target speech (i.e., the male speaker stream in the first half and the female speaker stream in the latter half) was reconstructed by the spatiotemporal filters *g*(τ,*n*) and the EEG responses *r*(*t*,*n*) at each electrode channel *n* over a range of time lag τ. The reconstructed speech envelope $\hat{s}\,\left(t\right)$ can be calculated in discrete time as


(3) $$\hat{s}\,\left(t\right)=\sum_{n}\sum_{\tau }r\,\left(t+\tau ,n\right)\,g\,\left(\tau ,n\right).$$

The linear mapping function *g*(τ,*n*) is estimated by ridge regression to avoid the overfitting and ill-posed problems, and the detailed procedure of ridge regression was referred to previous studies (e.g., Crosse et al., 2016). The leave-one-out cross-validation approach was implemented for optimizing the regularization parameter across subjects and conditions. Different regularization parameters searching from 2^0^, 2^2^, …, 2^12^ were used to reconstruct the auditory stimulus, respectively. The optimal regularization parameter was determined as 2^6^ because this value yielded the highest averaged correlation coefficient between the actual and reconstructed speech envelopes across the trained trials. The range of time lags was consistent with that contained in the major responses in the forward TRF, i.e., from 0 to 400 ms post-stimulus in this study.

After the processing of the leave-one-out cross-validation, the unified decoding model (*D*unified**) was used to predict the speech envelopes before and after attentional switching under different MFR conditions in the testing procedure. On the basis of the different effects of higher- and lower-RMS-level speech segments on cortical-envelope tracking ability to target speech streams, a segmented linear decoding model (*D*_*segmented*_) was proposed to separately reconstruct speech envelopes in higher- and lower-RMS-level segments, respectively (Wang, 2021). The decoder model of higher-RMS-level speech segments was generated by the EEG signals and auditory stimulus that only included higher-RMS-level segments. Similarly, lower-RMS-level speech segments and the corresponding EEG signals were used to train the specific model to decode lower-RMS-level speech segments. The training and validation procedures of these two decoders were the same as those used in *D*_*unified*_. In the testing procedure, the prior-trained SVM classifier was used to predict higher- and lower-RMS-level speech segments on the basis of the mixed speech and EEG responses. The speech envelopes were then reconstructed by the segmented decoders according to the boundaries of higher- and lower-RMS-levels speech segments. Finally, the reconstructed speech envelopes using *D*_*segmented*_ were generated by the concatenation of the predicted envelopes from different decoders. Subsequently, the AAD performance was determined by comparing the correlation coefficients between the reconstructed speech envelopes and the original envelopes of the target speech streams (*r*_*tar*_) or the ignored speech streams (*r*_*ign*_).

#### Performance of Auditory Attention Decoding

AAD accuracy was computed as the percentage of correctly identified trials (i.e., *r*_*tar*_ > *r*_*ign*_) in each condition. The AAD accuracies derived from *D*_*segmented*_ and *D*_*unified*_ were analyzed to show the effect of the attention switching between speakers under different MFR levels. The AAD accuracy could be an indicator to reveal the dynamic changes of the auditory attention states. In addition, to further test the sensitivity and reliability of the AAD systems, AAD accuracies were calculated with short to long decision window lengths (i.e., 1, 2, 5, 20, and 30 s) in different conditions. The Wolpaw information transfer rate (ITR) was used to assess the transmitted bits per time unit (Wolpaw and Ramoser, 1998). It was a metric that jointly evaluated the decoding accuracy and the decision time length of the AAD systems with different conditions. In this study, ITR was represented as bits per minute for five different decision window lengths τ (1, 2, 5, 20, and 30 s) with the AAD accuracy *p* of classification tasks. The detailed calculated equation was represented as


(4) $$I\,T\,R=\frac{1}{\tau }\,\left(1+p\,\log_{2}p+\left(1-p\right)\,\log_{2}\left(1-p\right)\right).$$

The effects of different decoding models, attention switching, and different MFR conditions on the ITR values were further statistically analyzed with the non-parametric Kruskal–Wallis test.

## Results

### Temporal Response Function Responses and Neural Encoding Performance

Repeated measures analysis of variance (ANOVA) was used to analyze the effects of the auditory attentional switching, RMS-level–based speech segments and the different SMR levels on TRF responses. Analyses of the magnitude of TRF responses in typical components were conducted by a 2 (attentional states: before vs. after attention switching) × 2 (speech feature: higher- vs. lower-RMS-level segments) × 3 (MFR level: –6 dB vs. 0 dB vs. 6 dB) within-subject repeated measures ANOVA. The Greenhouse–Geisser correction was adjusted the freedom when sphericity was violated, and the *post hoc* analysis was implemented with the Bonferroni correction to adjust *P*-value for multiple comparisons. Compared to the ignored speech stream, the target speech stream could elicit reliable and typical TRF components under various SMR conditions (e.g., Kong et al., 2014; O’Sullivan et al., 2015). Many studies also indicated that the TRF response obtained from the target speech streams contained biomarkers that could estimate the switching of the auditory attention states (e.g., Akram et al., 2016; Miran et al., 2020). Hence, this study showed and analyzed the typical TRF components elicited by the target speech streams in different conditions (see Figure 2B). TRF weights were statistically analyzed across three typical components within a specific window across all scalp electrodes (see Figure 2C).

For the first positive deflection, the average amplitude of the TRF weight was calculated from 80 to 150-ms time lags. ANOVA results revealed that a main effect for different RMS-level–based segments [*F*_(1, 15)_ = 16.77, *P* = 0.01, η${}_{p}^{2}$ = 0.53] and attention switching [*F*_(1, 15)_ = 22.43, *P* < 0.001, η${}_{p}^{2}$ = 0.60] with a significant interaction effect between these two factors [*F*_(1, 15)_ = 14.25, *P* = 0.002, η${}_{p}^{2}$ = 0.49]. These results suggested that the first positive components of the TRF response were larger with lower-RMS-level speech segments than with higher-RMS-level speech segments, and the TRF amplitudes in the first positive deflection were decreased after the switching of the auditory attention from one speaker stream to the other. There was no significant three-way interaction of different speech segments, attention switching, and MFR levels [*F*_(2, 14)_ = 0.58, *P* = 0.57, η${}_{p}^{2}$ = 0.08]. Neither the different speech segments by MFR level [*F*_(2, 30)_ = 3.00, *P* = 0.08, η${}_{p}^{2}$ = 0.30] nor the attention switching by MFR level interaction had significant effects on the amplitude of first positive deflection [*F*_(2, 30)_ = 0.80, *P* = 0.47, η${}_{p}^{2}$ = 0.10].

For the second positive deflection, the analysis window was set between 250 and 350 ms to compute the average amplitudes. The ANOVA results showed a main effect for different RMS-level–based segments [*F*_(1, 15)_ = 12.41, *P* = 0.003, η${}_{p}^{2}$ = 0.45] and MFR levels [*F*_(2, 30)_ = 10.29, *P* = 0.002, η${}_{p}^{2}$ = 0.60], indicating that the TRF amplitude of the second positive component was significantly larger with the lower-RMS-level segments than with higher-RMS-level segments, and this TRF weight was reduced with the decrease of MFR level. There was no main effect for attentional switching [*F*_(1, 15)_ = 0.97, *P* = 0.34, η${}_{p}^{2}$ = 0.06] suggesting that the TRF response around the 300-ms time lag was not significantly affected by the switching of attention in the competing speaker auditory scenes. No significant interactions were found with RMS-level–based speech segments, attention switching, and MRF level (all *P* > 0.05).

For the first negative deflection, the average TRF weight was computed within 170∼240 ms. The only significant main effect was revealed for the different RMS-level–based speech segments [*F*_(1, 15)_ = 13.79, *P* = 0.002, η${}_{p}^{2}$ = 0.48], showing the larger TRF responses in lower-RMS-level speech segments than those in higher-RMS-level speech segments. The attentional switching and MFR levels showed no main effects on the TRF amplitude of the first negative component (all *P* > 0.05). There were no significant three-way and two-way interactions of the three factors, i.e., RMS-level–based speech segments, attention switching, and MRF level (all *P* > 0.05).

### Classification of Higher- and Lower-Root Mean Square-Level Speech Segments

**FIGURE 3:**
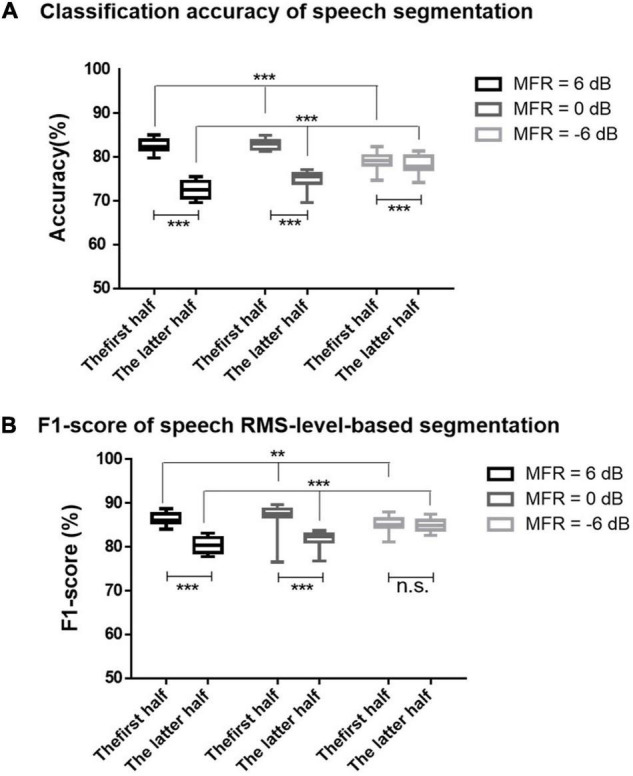
**(A)** The accuracies for the classification of higher- and lower-RMS-level speech segments using SVM classifier under 6-dB (black signs), 0-dB (dark gray signs), and –6-dB (light gray signs) MFR conditions before and after the switching of the auditory attention at the middle time of the 60-s trails. **(B)** The F1-scores for the classification of higher- and lower-RMS-level speech segments under 6-dB (black signs), 0-dB (dark gray signs), and –6-dB (light gray signs) MFR conditions in the first half and the latter half. The bars represent the max and min values of each condition, *** indicates *P* < 0.001, ** indicates *P* < 0.01, and n.s. indicates *P* > 0.05.

On the basis of the different neural patterns for higher- and lower-RMS-level speech segments of the target speech perception under noisy environments, the current study utilized the corresponding cortical responses to predict the higher- and lower-RMS-level speech segments of the auditory speech stimuli. Figure 2A displays the RMS level of a whole sentence, and the dashed line indicates the RMS threshold to determine higher- and lower-RMS-level segments. By averaging the percentages of all sentences used in this experiment, the duration of higher- and lower-RMS-level segments accounted for 51.22 and 48.78% of the whole utterances, respectively, which was consistent with the previous findings that the higher- and lower-RMS-level segments had similar duration within the continuous sentences (Chen and Loizou, 2011; Wang, 2021). The higher-RMS-level speech segments comprised 57.81, 69.43, and 59.66% durations of mixed speech under the 6–, 0–, and –6-dB MFR conditions, respectively. The classified results of higher- and lower-RMS-level speech segments were calculated with the short time fragments using the trained SVM classifier. Figure 3 shows the classification accuracy and F1-score of higher- and lower-RMS-level speech segments before and after the attentional switching from male to the female speaker stream under different MFR levels. The effect of attention switching and MFR level on the SVM classification results were examined with the non-parametric Kruskal–Wallis test. There were significant effects of attention switching and MFR level on the classification accuracy of different speech segments (all *P* < 0.001). Specifically, the classification accuracy was decreased after the switching of the auditory attention from the male speaker to the female speaker with the 6-dB MFR (the first half: mean = 82.50, standard error = 0.46; the latter half: mean = 72.47, standard error = 0.46), the 0-dB MFR (the first half: mean = 81.73, standard error = 1.10; the latter half: mean = 78.13, standard error = 0.48), and the –6-dB MFR (the first half: mean = 79.37, standard error = 0.36; the latter half: mean = 74.73, standard error = 0.50). These results indicated that the classification accuracy was significantly affected by the auditory attentional switching with a wide range of MFR conditions (i.e., from 6 to –6 dB). The F1-scores in the first 30 s were higher than those in the latter half with the effect of attention switching under the 6-dB MFR (the first half: mean = 86.34, standard error = 0.34; the latter half: mean = 80.34, standard error = 0.43) and the 0-dB MFR (the first half: mean = 87.19, standard error = 0.72; the latter half: mean = 81.68, standard error = 0.43). No significant differences of the F1-score were shown before and after the attention switching between two speaker streams under the –6-dB MFR [(χ^2^ = 1.20, *P* = 0.27); the first half: mean = 85.46, standard error = 0.29; the latter half: mean = 84.87, standard error = 0.36]. Both classification accuracy and F1-score were reduced with the decreased SMR levels in the first half and the latter half (all *P* < 0.01), suggesting that the relative SMR level was a critical factor to influence the classification performance of higher- and lower-RMS-level speech segments from the EEG signals.

### Auditory Attention Decoding Performance

#### Correlation Coefficients Between Actual and Predicted Speech Envelopes

**FIGURE 4:**
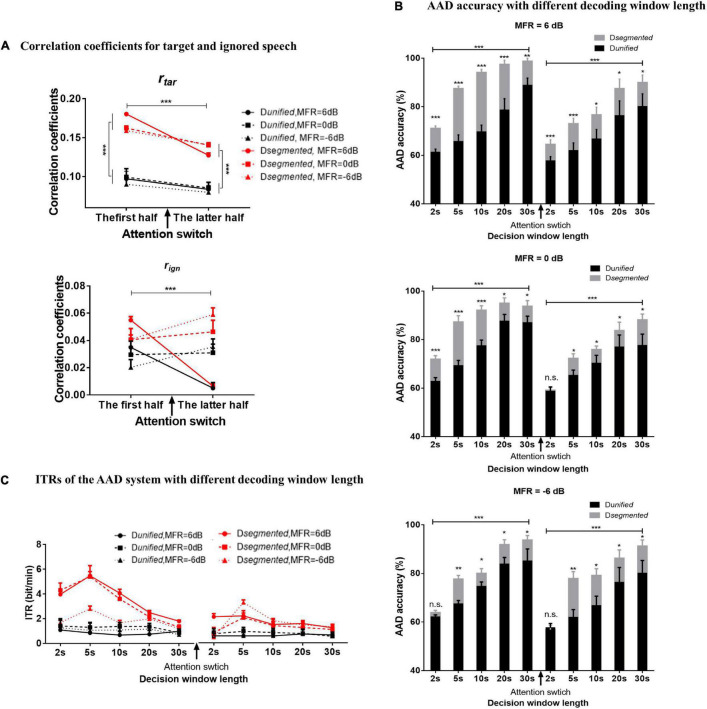
**(A)** The correlation coefficients of the reconstructed speech envelopes with the target speech envelope (the above figure) and the ignored speech envelopes (the below figure) decoded with the *D*_*segmented*_ (red lines) and *D*_*unified*_ (black lines) computational models in 6-dB (solid lines with circle signs), 0-dB (dashed lines with square signs), and –6-dB (dot lines with rectangular signs) MFR conditions in the first half and the latter half. **(B)** The AAD accuracy calculated by *D*_*segmented*_ (gray boxes) and *D*_*unified*_ (black boxes) with 2–, 5–, 10–, 20–, and 30-s decoding window lengths before and after the switching of auditory attention in 6–, 0–, and –6-dB MFR conditions. The error bars show the standard error in each condition, *** indicates *P* < 0.001, ** indicates *P* < 0.01, * indicates *P* < 0.05, and n.s. indicates *P* > 0.05. **(C)** ITR with the *D*_*segmented*_ (red lines) and *D*_*unified*_ (black lines) with 2, 5, 10, 20, and 30 s decoding window lengths before and after the switching of the auditory attention in 6–, 0–, and –6-dB MFR conditions.

Figure 4A shows the correlation coefficients between the reconstructed and original speech envelopes to the target or ignored speech before and after the attention switching under the 6–, 0–, and –6-dB MFR conditions using *D*_*unified*_ and *D*_*segmented*_, respectively. The decoding window length of 30 s was used to calculate the *r*_*tar*_ and *r*_*ign*_ values in Figure 4A, and the relative value of *r*_*tar*_ and *r*_*ign*_ was the basis for determining the attentional direction in the competing speaker scenes. The ANOVA analysis showed a main effect for the type of reconstructed speech streams, showing that *r*_*tar*_ was significantly larger than *r*_*ign*_ [*F*_(1, 15)_ = 93.35, *P* < 0.001, η${}_{p}^{2}$ = 0.86] under all experimental conditions in this study. A three-way ANOVA analysis was also performed to test the effects of different decoding models, MRF levels, and attentional switching on *r*_*tar*_ and *r*_*ign*_ values, respectively. There were no significant interactions of these three factors, and the interaction of decoding model by MFR level for both *r*_*tar*_ and *r*_*ign*_ values (all *P* > 0.05). A significant interaction was shown between MFR level and attention switching for the value of *r*_*tar*_ [*F*_(2, 30)_ = 5.19, *P* = 0.01, η${}_{p}^{2}$ = 0.26] and *r*_*ign*_ [*F*_(2, 30)_ = 28.01, *P* < 0.001, η${}_{p}^{2}$ = 0.65]. The attention switching exhibited a main effect on the value of *r*_*tar*_ [*F*_(1, 15)_ = 43.03, *P* < 0.001, η${}_{p}^{2}$ = 0.74], but no significant main effect for MFR level on the value of r*_*tar*_* [*F*_(2, 30)_ = 0.70, *P* = 0.50, η${}_{p}^{2}$ 0.05]. For the value of *r*_*ign*_, both attention switching [*F*_(1, 15)_ = 43.03, *P* < 0.001, η${}_{p}^{2}$ = 0.74] and MFR level [*F*_(2, 30)_ = 0.70, *P* = 0.004, η${}_{p}^{2}$ = 0.31] showed significant main effect. *Post hoc* analysis showed that the *r*_*tar*_ values in the latter half were significantly smaller than those in the first half with the 6–, 0–, and –6-dB MFR conditions. The changes of the *r*_*ign*_ values after attention switching from male to female speaker streams were dependent on the SMRs, i.e., no significant differences in 0-dB MFR condition, a decrease of *r*_*ign*_ value with the SMR reduce (i.e., the 6-dB MFR condition), and increased *r*_*ign*_ value with the increase of SMR levels (i.e., the –6-dB MFR condition). These results suggested that the *r*_*tar*_ values were robustly modulated by auditory attention, and the attentional gains controlled the reliable cortical responses to target speech streams regardless of the relative intensity of the competing streams in a wide range of SMR conditions (i.e., 6 to –6 dB in this study), whereas the *r*_*ign*_ values showed significant effects of the SMR changes with attentional switching. In addition, the main effect was significant for different decoding models in both *r*_*tar*_ [*F*_(2, 30)_ = 5.19, *P* = 0.01, η${}_{p}^{2}$ = 0.26] and *r*_*ign*_ values [*F*_(2, 30)_ = 28.01, *P* < 0.001, η${}_{p}^{2}$ = 0.65], revealing that the RMS-level–based *D*_*segmented*_ improved the reconstructed performance of speech envelopes than the *D*_*unified*_.

#### Auditory Attention Decoding Accuracy and Sensitivity

To examine the AAD performance of the neuro-steered system with different decoding algorithms (i.e., *D*_*unified*_ and *D*_*segmented*_) before and after the attentional switching from the male to the female speaker stream, the non-parametric Kruskal–Wallis test was implemented to analyze the AAD accuracy with different decision window lengths (i.e., 2, 5, 10, 20, and 30 s). Figure 4B and Table 1 show the detailed AAD accuracies under different experimental conditions. The AAD accuracies using *D*_*segmented*_ were significantly higher than those using *D*_*unified*_ under all experimental conditions (all *P* < 0.05), except for the conditions where the decoding window length was 2 s with the 0-dB MFR after attention switching and with the –6-dB MFR before and after attention switching between two speech streams (*P* > 0.05). The AAD accuracy was significantly increased with the extension of decision window time before and after the auditory attention switching with three MFR conditions (all *P* < 0.05). In both the 6- and 0-dB MFR conditions, the AAD accuracies were significantly reduced after attention switching using both the *D*_*unified*_ and *D*_*segmented*_ (all *P* < 0.05), suggesting that the switching of the auditory attention in the competing speaker scenes affected the AAD performance. There was a marginal decrease of AAD accuracy after the switching of attention from the male to the female speaker stream with the –6-dB MFR condition using the five decoding window lengths, indicating that the increased SMR level could supplement the decrease of AAD accuracy after attention switching.

**TABLE 1:** The averaged AAD accuracies and the standard deviations (mean/standard deviation) decoded by *D*_*unified*_ and *D*_*segmented*_ using different decoding window length (i.e., 2, 5, 10, 20, and 30 s) before and after the switching of attention from the male to the female speaker streams under the 6–, 0–, and –6-dB MFR conditions.

Decoding window length		MFR = 6 dB		MFR = 0 dB		MFR = –6 dB	
		The first half	The latter half	The first half	The latter half	The first half	The latter half
*D*_*unified*_	2 s	61.17/1.37	57.58/1.76	62.71/1.62	58.63/1.85	62.04/0.86	57.58/1.84
	5 s	65.63/2.78	61.88/3.03	69.17/2.26	65.21/2.21	67.40/1.39	61.88/3.21
	10 s	69.58/2.82	66.67/3.74	77.40/2.52	70.21/3.31	74.58/1.85	66.67/3.95
	20 s	78.57/4.84	76.25/5.49	87.50/2.86	76.88/4.98	85.00/2.79	76.25/6.18
	30 s	88.75/3.04	80.00/6.17	86.86/2.76	77.50/4.72	88.00/3.16	80.00/5.30
*D*_*segmented*_	2 s	71.08/0.94	64.50/1.87	71.92/1.38	59.17/0.99	63.89/0.86	56.83/1.81
	5 s	87.50/0.93	73.04/2.20	87.29/2.41	72.29/1.36	77.69/1.39	77.91/2.69
	10 s	94.17/1.09	76.67/3.12	92.08/1.77	75.88/1.77	80.04/1.85	79.17/2.69
	20 s	97.50/1.56	87.50/3.90	95.00/2.06	83.75/2.28	93.75/2.66	86.25/3.23
	30 s	98.75/1.14	90.00/3.06	93.75/2.21	88.13/3.21	91.88/1.71	91.25/2.94

The ITRs were also statistically analyzed to assess the sensitivity of the AAD system using the non-parametric Kruskal–Wallis test. Figure 4C displays the effect of attention switching, different decoding models (*D*_*unified*_ and *D*_*segmented*_), and different MFR levels on the ITRs. The *D*_*segmented*_ model yielded higher ITRs than the *D*_*unified*_ model before and after the switching of the auditory attention with all MFR levels (*P* < 0.05), suggesting the significant improvement of AAD accuracy based on the speech-RMS-level–based decoding model. Significantly higher ITRs were displayed with the 6- and 0-dB MFR conditions than the –6-dB MFR level in the first half (i.e., before the attention switching). *Post hoc* analysis showed that the significant differences occurred with the short decision window lengths (i.e., 2, 5, and 10 s; all *P* < 0.01). In the latter half (i.e., after the switching of attention), a significantly higher ITR was shown in the 6-dB MFR than the 0- and –6-dB conditions with 2-s length of the decoding decision window (χ^2^ = 7.02, *P* = 0.03). There were no significant differences in ITRs across the five decision window lengths under all MFR conditions using *D*_*unified*_ (all *P* > 0.05). For the effect of attention switching, there were significant decreases of ITRs with the 6- and 0-dB MFRs after the switching of the auditory attention between two competing speakers using *D*_*segmented*_ (all *P* < 0.05). In the –6-dB MFR condition, the attention switching had no significant effect on ITR decoded by *D*_*segmented*_ (χ^2^ = 1.33, *P* = 0.25). No significant effects of attention switching were shown with the *D*_*unified*_ model in all three MFR conditions (all *P* > 0.05).

## Discussion

The present study aimed to develop objective biomarkers on the basis of the neural-speech tracking ability to estimate the dynamic auditory attention states under the competing speaker auditory scenes. The present study also explored the effects of the RMS-level–based speech segmentation and SMR level on the AAD performance with the dynamic change of attention states. This work provided several important and novel findings for better understanding the neural mechanisms of the target speech perception in the complex auditory scenes. First, the switching of the auditory attention from one speaker stream to the other can be detected from the corresponding EEG responses with short time lags (i.e., the first TRF-positive deflection approximately 100 ms). Second, the cortical tracking ability to the target speech was different between higher- and lower-RMS-level–based speech segmentations. On the basis of these different neural responses, the RMS-level–based segmented model improved the accuracy and sensitivity of the neuro-steered AAD system. Third, the SMR level and attentional states (before or after the attentional shifting) jointly affected the attention decoding performance in the competing speaker auditory scenes. The robust AAD accuracy was shown with a wide range of SMR levels, and the AAD accuracy was also sensitive to the switching of the auditory attention.

### Effect of Root Mean Square-Level–Based Segmentation on Decoding Auditory Attention States

In line with previous findings (e.g., Wang et al., 2019, 2020a), this study also showed significantly different neural responses to higher- and lower-RMS-level speech segments when subjects concentrated their attention on one of the speaker streams in the competing speaker conditions. Significantly higher TRF weights were shown in lower-RMS-level speech segments than those in higher-RMS-level speech segments, indicating high correlations between neural responses and speech envelopes in lower-RMS-level segments. These results implied that the total energy of neural response evoked by lower-RMS-level speech segments was stronger than that by higher-RMS-level speech segments. Not only the relative RMS level but also the speech features carried in higher- and lower-RMS-level speech segments could be contributing factors to the target speech perception in noisy environments. More specifically, higher-RMS-level speech segments contained most voicing parts of the whole utterance, whereas lower-RMS-level speech segments carried most changeable components such as the abrupt increases and decreases sections of the whole utterance (e.g., Chen and Loizou, 2011, 2012; Chen and Wong, 2013). The large TRF responses with lower-RMS-level speech segments were consistent with the previous findings that the cortical responses were sensitive to the abrupt changes within the auditory stimulus (Chait et al., 2005; Somervail et al., 2021).

Furthermore, this study found that the switching of the auditory attention had different effects on the cortical responses to higher- and lower-RMS-level speech segments. After the switching of attention from the male to the female speaker stream, the significant decrease of the first positive components in the TRF responses (approximately 100-ms time lag) was illustrated for both higher- and lower-RMS-level speech segments. These results were consistent with previous findings in ERP studies that the early component (e.g., P100) was related to the attention-dependent modulation (Shuai and Elhilali, 2014). Although lower-RMS-level speech segments showed stronger TRF weights than higher-RMS-level speech segments for all three typical components, attention switching showed no significant modulations of the cortical responses to lower-RMS-level speech segment in the first negative and second positive TRF components. Besides, the TRF weights with lower-RMS-level speech segments were sensitively changed with the SMR levels. These results suggested that the lower-RMS-level segments were easily affected by the environmental factors (e.g., the intensity of the competing speech stream) (Billings et al., 2009). Compared to cortical response to lower-RMS-level speech segments, the TRF responses with higher-RMS-level segments were robust to the SMR level changes and sensitive to the modulation of the auditory attention. Higher-RMS-level speech segments that included more complex speech cues (e.g., semantic information and language structures) could be primarily influenced by the modulation of endogenous factors (e.g., selective auditory attention) rather than exogenous variables (e.g., SMR levels) (Getzmann et al., 2017). Briefly, this study demonstrated that, under the dynamic auditory attention states, the auditory system recruited different neural response patterns to track higher- and lower-RMS-level speech segments under different SMR conditions.

The effects of RMS-level–based segmentation on the AAD performance were further explored on the basis of the different neural responses to higher- and lower-RMS-level speech segments with the dynamic changes of attentional states. According to our previous investigation, the speech-RMS-level–based segmented AAD model could improve AAD sensitivity and accuracy when subjects were concentrated on a specific speech stream during the whole experiment (Wang, 2021). This study further demonstrated that the segmented AAD model not only improved the AAD accuracy under the conditions modulated by the sustained attention, but also improved the AAD accuracy when attention was transferred from one speech stream to the other in a competing speaker environment (see Figure 4B). The better performance of the segmented AAD model could be attributed to the accurate detection of temporal gaps, because the temporal gaps in continuous sentences can facilitate the target speech perception in noisy environments (e.g., Li and Loizou, 2007; Vestergaard et al., 2011). Many neurological studies also suggested that the regular structure of temporal gaps within the continuous sentences entrained the low-frequency neural oscillations to track the target speech streams with the selective attention modulations (Hickok and Poeppel, 2007; Zoefel, 2018). Correspondingly, lower-RMS-level speech segments contained the temporal gaps (i.e., the silent regions) and weak consonants (e.g., fricatives, stops, and nasals) of a sentence, whereas higher-RMS-level speech segments carried most sonorous parts within an utterance (Chen and Loizou, 2011; Chen and Wong, 2013). Hence, the prior knowledge of speech-RMS-level segmentation provided much detailed temporal information of speech, so that the *D*_*segmented*_ method could decode the target speech streams more accurately from neural activities. The AAD accuracy calculated by the *D*_*segmented*_ method was not only affected by the reconstructed performance of target speech envelopes but also associated with the classification performance of higher- and lower-RMS-levels segments under different experimental conditions. As displayed in Figure 3, the classification accuracy of higher- and lower-RMS-level speech segments was decreased with the attention switching from the male to the female speaker stream. When the auditory attention was switched between competing speakers, neural resources related to the target auditory object needed to be redistributed through the modulation of selective auditory attention (e.g., Fritz et al., 2007; Shamma and Micheyl, 2010). Because the auditory system was required to release the resources related to the prior focused streams and active the resources belonging to the switched auditory objects, a weak gain of the attention modulation could occur and lead to the poor neural tracking ability after the switching of attention (e.g., Getzmann et al., 2017; Miran et al., 2018). Hence, the AAD accuracy was reduced after the auditory attention switching from the male to the female speaker stream. This study indicated that the speech-level–based segmented decoding model not only had better AAD performance with the sustained auditory attention but also improved the AAD performance after the switching of the auditory attention in the complex auditory scenes. These results provided evidence that the segmented AAD model had the potential to decode auditory attention in real-life applications with the dynamic change of attention states.

### Interactions Between Attention Switching and Signal-to-Masker Ratio Levels on the Auditory Attention Decoding System

In a competing speaker environment, the SMR level is an important factor affecting the target speech perception, and the target speech intelligibility is reduced with the decrease of SMR levels (Brungart, 2001; Billings et al., 2009). Nevertheless, the cortical responses showed the robust phase locking of the target speech envelopes with a large range of SMR levels (e.g., Ding and Simon, 2012b; O’Sullivan et al., 2015). These reliably cortical responses to the target speech envelope were associated with the attentional gain control and the long-term integration of the slow temporal modulations in the human auditory cortex (Lalor et al., 2009; Kerlin et al., 2010). In line with previous studies (e.g., Ding and Simon, 2012b; Di Liberto et al., 2015; O’Sullivan et al., 2015), this study also suggested that the neural responses were reliably synchronized to slow temporal fluctuations of the target speech with the sustained attention under different SMR conditions (i.e., from 6 to –6 dB). However, it still remained unclear about the effect of attention switching on the AAD performance under diverse SMR conditions. Studies have illustrated the effect of attention switching between the co-located competing speakers with the equal RMS levels of sound amplitude, suggesting that the TRF response carried effective biomarkers to estimate the auditory attention states (e.g., Akram et al., 2016; Miran et al., 2018, 2020). On the basis of these findings, the current study further explored the joint effect of the attention switching and SMR levels on the AAD performance without the spatial difference between speakers. To evaluate the AAD ability with attention switching from moderate to severe SMR conditions, the relative power ratios between male and female speaker streams were fixed in this study, and thus, the SMR level could change with the attention switching from the male to the female speaker stream. Results demonstrated that the cortical responses can be used to decode the switching of the auditory attention with the increased SMRs (from –6 to 6 dB SMR in the –6-dB MFR condition), the unchanged SMRs (in the 0-dB MFR condition) within the continuous speech streams, and the decreased SMRs (from 6 to –6 dB SMR in the 6-dB MFR condition). The marginal decrease of AAD accuracy was displayed after the switching of the auditory attention in all three MFR conditions (see Table 1 and Figure 4B). It may be associated with the cost of attention switching. Compared to the condition with decreased and unchanged SMRs after attention switching, the increased SMR could alleviate the decrease of AAD accuracy with the switching of attention between two speakers. The AAD accuracy after the switching of the auditory attention also showed the larger individual differences than that before the auditory attention switching. These individual differences implied that the AAD performance with the dynamic changes of auditory states may be related to some endogenous factors such as the attentional control gains and the predicting ability of important cues in the target speech (Kerlin et al., 2010; Getzmann et al., 2017), which warrants further investigation in the future.

### Objective Neural Markers of Auditory Attention States

Neuroimaging studies using magneto-encephalography have illustrated that the magnitude of the TRF component approximately 100-ms lag was a reliable attention marker, because the TRF responses at 100-ms lag of the target speaker were larger than those of the ignored speaker (Ding and Simon, 2012a; Akram et al., 2016; Miran et al., 2020). In this study, the TRF responses obtained from EEG signals also showed a reliable marker modulated by the switched auditory attention with latency approximately 100-ms lag. Specifically, compared to the other typical TRF components, the TRF weight at the first positive component showed reliable effects of attention switching for both higher- and lower-RMS-level speech segments with a large range of SMR levels (i.e., from –6 to 6 dB) in this study. The observed changes of the TRF component approximately 100-ms lag with the attention switching were in agreement with previous findings in ERP studies that the peak of the P1 component was modulated by purely top-down attention and marked the initiation of a new auditory stream of the ongoing stream (Winkler et al., 2009; Shuai and Elhilali, 2014). These results suggested that the encoder model not only reflected the precision of neural tracking ability to the target speech but also provided the objective biomarker to index the dynamic attention states (e.g., before and after the switching of attention). In addition, the present study revealed the decrease of AAD accuracy after the auditory attention switching (see Table 1), suggesting the fluctuation of AAD accuracy may also be an indicator to estimate the switching of the auditory attention in a competing speaker environment. The *D*_*segmented*_ method showed higher ITRs than the *D*_*unified*_ method in the neural-based AAD system, especially with the short decoding window length (i.e., 2, 5, and 10 s) in various experimental conditions. The better performance of the segmented model with short decision window lengths suggested that the AAD accuracy derived from the *D*_*segmented*_ decoder could also be an effective indicator to evaluate the dynamic change of the auditory attention states.

### Limitations of This Work

This study mainly explored the joint effects of the auditory attention states, SMRs, and higher/lower-RMS-level–based segments on cortical responses to the target speech streams, and the AAD performance decoded by the speech-level–based segmented computational model was investigated under different experimental conditions. Hence, other crucial characteristics of the competing speakers were fixed in this experiment. Specifically, this study only examined the switching of the auditory attention from the male speaker to the female speaker under different MFR conditions. Nevertheless, cortical responses are influenced by a number of voice characteristics (e.g., fundamental frequency differences between the competing speakers) in the complex auditory scenes (e.g., van Canneyt et al., 2021). Further research should systemically understand the effects of other features (e.g., speaker gender, number of speakers, and target-to-masker ratios) on the cortical tracking ability of the target speech streams in the complex auditory scenarios with the dynamic changes of the auditory attention.

## Conclusion

This study investigated the effects of different RMS-level–based speech segments and SMR levels on the cortical tracking ability to the target speech with sustained and switched auditory attention. The present study also explored effective objective indicators for reflecting dynamic attention states from EEG recordings under the competing speaker environments. The novel findings in this study included the following: (a) the TRF response at 100-time lag could sensitively index the switching of the auditory attention from one speaker stream to the other; (b) higher- and lower-RMS-level speech segments made different and crucial contributions to the cortical tracking of the target speech with both the sustained and switched auditory attention. On the basis of the specific neural patterns to different RMS-level segmentation, the segmented AAD model, which provided more exact temporal structures of the target speech, improved the AAD performance of dynamic attention states; (c) the segmented AAD model could be used to robustly decode the dynamic changed target speech streams according to their intentions under different SMR conditions, even when using a short decoding window length.

In conclusion, TRF responses and AAD accuracies could be considered as objective indicators for estimating the auditory attention states even in poor SMR conditions and with short decision window lengths. The RMS-level–based segmented AAD model also showed the sensitive and reliable decoding performance with the attentional switching. Results exhibited in this work provided neural evidence for understanding the contributions of different speech features on cortical response to the target speech with the dynamic modulation of the auditory attention. These results also provided potential guidance for the design of AAD algorithms in the neurofeedback control systems under complex auditory scenarios.

## Data Availability Statement

The raw data supporting the conclusions of this article will be made available by the authors, without undue reservation.

## Ethics Statement

The studies involving human participants were reviewed and approved by the Institution’s Ethical Review Board of Southern University of Science and Technology approved the experimental procedures. The patients/participants provided their written informed consent to participate in this study.

## Author Contributions

LW contributed to the design and implementation of the experiments, the analysis and interpretation of data, and the writing of the manuscript. YW and ZL performed data acquisition. EW contributed to the revision of the manuscript and final approval of the submitted version. FC contributed to the design of experiments, the interpretation of data, the revision of the manuscript, and the final approval of the submitted version. All authors contributed to the article and approved the submitted version.

## Conflict of Interest

The authors declare that the research was conducted in the absence of any commercial or financial relationships that could be construed as a potential conflict of interest.

## Publisher’s Note

All claims expressed in this article are solely those of the authors and do not necessarily represent those of their affiliated organizations, or those of the publisher, the editors and the reviewers. Any product that may be evaluated in this article, or claim that may be made by its manufacturer, is not guaranteed or endorsed by the publisher.
